# Cervical yolk sac tumor: a case report and literature review

**DOI:** 10.3389/fonc.2026.1782181

**Published:** 2026-03-12

**Authors:** Ming Wang, Shuiqing Xu, Yang Zhan, Bei Fan, Yumei Wu

**Affiliations:** 1 Department of Gynecologic Oncology, Beijing Obstetrics and Gynecology Hospital, Capital Medical University, Beijing Maternal and Child Health Care Hospital, Beijing, China; 2 Department of Pathology, Beijing Obstetrics and Gynecology Hospital, Capital Medical University, Beijing Maternal and Child Health Care Hospital, Beijing, China

**Keywords:** alpha-fetoprotein, antineoplastic combined chemotherapy protocols, cervix neoplasms, germ cell tumors, yolk sac tumor

## Abstract

**Background:**

Yolk sac tumor (YST) is the third most common highly malignant germ cell tumor, predominantly arising from the ovary and testis. The standard treatment for ovarian YST is surgery combined with BEP (bleomycin, etoposide, and cisplatin) chemotherapy in which bleomycin has a cumulative lifetime dose and pulmonary toxicity. Advancements in treatment have yielded favorable prognoses for YST patients. Here, we present the clinical, imaging, pathological features, and individualized treatment of a case of primary cervical YST which is extremely rare in previous reports.

**Case report:**

A 46-year-old female patient with primary cervical YST received neoadjuvant chemotherapy(1 cycle of paclitaxel+carboplatin, 2 cycles of BEP), radical surgery and 4 cycle of adjuvant BEP chemotherapy. The patient achieved complete remission after initial treatment but developed disease recurrence shortly thereafter, with confirmed BEP resistance. Salvage treatment with TC (albumin-bound paclitaxel and carboplatin) combined with bevacizumab was then administered, leading to a durable complete response; the patient subsequently received bevacizumab as maintenance therapy.

**Conclusions:**

YST of the cervix is extremely rare. This article reviews the clinical characteristics, treatment, and survival of advanced cervical YST. In addition, the complete response of this patient provides a new option for patients with recurrent and metastatic cervical YST, and TC regimen combined with VEGF inhibitors is expected to serve as a salvage treatment.

## Background

Yolk sac tumor (YST) is a relatively rare malignant germ cell tumor, most commonly found in infants and young women, and rarely in women over 40 years of age. It usually originates from the gonads (ovaries, testicles) (1–3) or is distributed along the central axis of the body (4–8). About 10 to 20% of YSTs originate in extragonadal sites, most commonly in sacrococcygeal region, mediastinum, vagina, omentum, pineal gland, and retroperitoneum (4–8). The pathogenesis of YSTs outside the gonadal gland is still unclear, although it is generally accepted to be a result from the abnormal migration of germ cells during embryogenesis or differentiation of abnormal somatic cells (6, 7).

Most of the symptoms of a YST come from the rapid growth or enlargement of the tumor pressing on the surrounding organs. For example, most patients with ovarian YSTs manifested abdominal distention and sudden onset of pelvic or abdominal pain (1). A pelvic mass may be palpable on examination. Patients with vaginal or cervical YST may manifest as irregular vaginal bleeding (9, 12). The diagnosis of YSTs can be considered based on history, physical examination, imaging, and tumor markers. Alpha fetoprotein (AFP) is the specific tumor marker for YST which could be used for diagnosis and evaluation of treatment response (1). Positive immunohistochemical staining of AFP can support the diagnosis of YSTs for nearly all cases stain positive for AFP. This YST case manifested as an enlarged cystic and solid mass with intratumoral hemorrhage on CT and MRI (12).

YST of the cervix is extremely rare and mainly occurs in infants younger than 3 years old (9–14). Mardi K, et al. have reported a case of a cervical YST in an adult, although detailed information on the treatment and prognosis was not included (12) (**Table 1**). Currently, there is no standard treatment for cervical YST in the practical guidelines due to its rarity. Here, we present the clinical characteristics and treatment information of a case of cervical YST in an adult patient.

**Table 1:** Previous report of cervical yolk sac tumor.

Author (year)	Numbers of case	Age	Tumor site	Treatment regimen	Follow-up duration	Clinical prognosis
Chen SJ, et al. (1992)	1	6 months	vagina and cervix	surgery	NA	NA
Narasimhan S, et al. (2022)	1	5 months	cervix and lower uterine	Surgery and chemotherapy	18 months	Alive without relapse
Torino G, et al. (2021)	1	11 months	uterine cervix	Surgery and chemotherapy	12 months	Alive without relapse
Mardi K, et al. (2022)	1	20 years	Cervix and vagina	surgery	NA	NA
Yadav K et al. (1996)	1	NA	cervix	NA	NA	NA
Copeland LJ, et al. (1985)	6	All were younger than 3 years old	Cervix and vagina	Five received surgery combined with chemotherapy; 1 received chemotherapy	2 to 23 years	1 had pulmonary metastasis

## Case report

**Figure 1:**
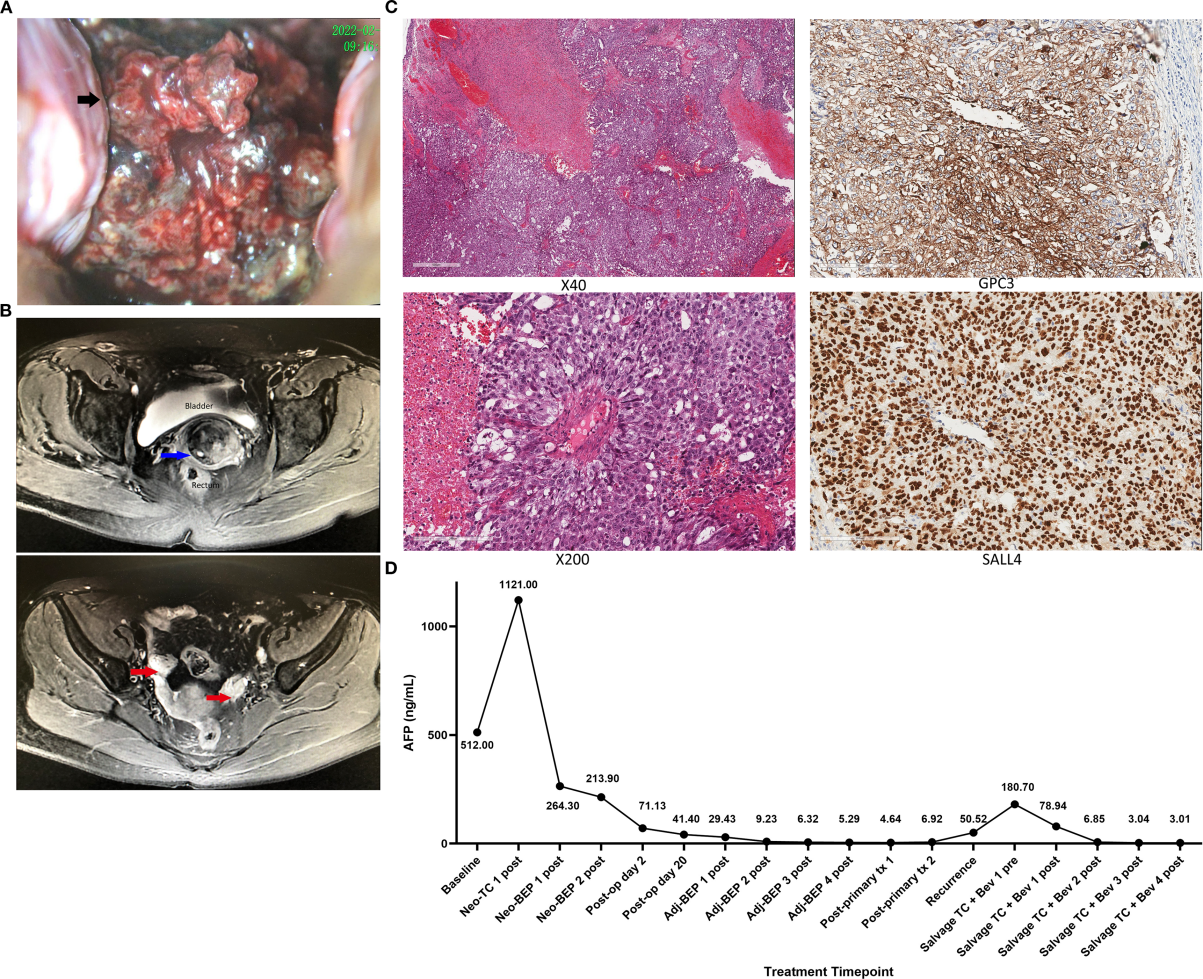
**(A)** Vaginal examination: an 8cm cauliflower-like cervical mass with a friable surface and prone to bleeding and necrosis. **(B)** Enhanced MRI scans: a solid mass (approximately 5.3 x 2.7 x 6.4 cm in size) located in the exocervix (blue arrow) and no lesions were observed in the bilateral ovaries (red arrow). **(C)** The biopsy of the cervical tumor: under microscopy (magnification:x40;x200), tumor cells were distributed solidly with lumens of varying sizes and arranged in the periphery around some blood vessels. Immunohistochemical staining for SALL4, GPC3, LIN28, and AFP was positive. **(D)** Serial Serum AFP Monitoring During Neoadjuvant, Adjuvant and Salvage Therapy for Recurrent Cervical Yolk Sac Tumor. Baseline, Pre-treatment AFP level; Neo, Neoadjuvant; TC, Albumin-bound paclitaxel + carboplatin; BEP, Bleomycin, etoposide, cisplatin; Adj, Adjuvant; Post-op, Post operation; Pre, Pre-treatment; Post, Post-treatment; Salvage, Salvage therapy; Bev, Bevacizumab; tx, Treatment.

A previously healthy 46-year-old woman presented to Beijing Obstetrics and Gynecology Hospital (Beijing, China) with unprovoked intermittent vaginal bleeding for 9 months. She had an unremarkable personal and family medical history, with no malignant or genetic diseases reported in first-degree relatives. She had a regular menstrual pattern for years: menarche at 14 years old, a 28–30-day cycle, 5–6 days of menstruation per cycle, moderate flow, and no dysmenorrhea. She had no history of sexual activity, pregnancy, childbirth, gynecological surgery, or hormone medication use. Vaginal examination revealed an 8cm cauliflower-like cervical mass with a friable surface prone to bleeding and necrosis (**Figure 1A**). Serum AFP concentration was 512 ng/mL at baseline (normal range 0.0–10.0 ng/mL). Enhanced MRI and CT scans revealed a solid mass located in the exocervix (approximately 5.3 x 2.7 x 6.4 cm in size) and an additional metastatic lymph node near the left iliac vessel with a short-axis diameter of 18 mm (**Figure 1B**).

No abnormalities were identified on imaging in the ovaries, peritoneum, other pelvic or abdominal organs. The biopsy of the cervical tumor identified a poorly differentiated malignant tumor with hemorrhage and necrosis. Under light microscopy (magnification: ×200), the tumor cells formed a solid pattern with scattered variable-sized gland-like/cystic lumens; partial tumor cells arranged in a perivascular radial pattern (Schiller-Duval body-like structure) around the small blood vessels, which is a typical morphological feature of yolk sac tumor. Immunohistochemical staining for SALL4, GPC3, LIN28, and AFP was positive, leading to a diagnosis of a YST (**Figure 1C**). The patient was diagnosed with a rare primary cervical YST stage IIIC1 according to the International Federation of Gynecology and Obstetrics (2018).

The treatment plan of neoadjuvant chemotherapy followed by cytoreductive surgery was made based on doctor-patient’s decision extrapolated from the treatment modality for YSTs of the ovary and the anatomic characteristics of this tumor. The patient received 3 cycles of neoadjuvant chemotherapy (NACT) (1 cycle of paclitaxel and carboplatin before immunohistochemistry and two cycles of bleomycin, etoposide, and cisplatin, as per the standard of care for YST) until tumor size reduction and a partial response were achieved (AFP decreased to 213.9 ng/ml and the cervical tumor decreased to 4.1 x 4.3 x 4.1 cm on CT scan). Then a radical hysterectomy, bilateral salpingo-oophorectomy, retroperitoneal lymphadenectomy, and omentectomy were performed. On the second postoperative day, AFP had significantly decreased to 71.13 ng/ml. Postoperative pathology revealed high-risk pathological and clinical features associated with elevated recurrence risk:the tumor infiltrated one to two thirds of the cervical wall (1.5 cm thickness), microscopic residual tumor tissues were seen in the exocervix, right sacral ligament, and left ovary, and lymphovascular space invasion (LVSI) of the tumor was seen in both the cervix and left ovary. Confirmed left external iliac lymph node metastasis further defined the patient as FIGO stage IIIC1 (2018), a late-stage classification for gynecologic malignancies with significantly increased recurrence potential. After the surgery, she received 4 cycles of adjuvant BEP chemotherapy, with AFP normalization (9.23 ng/mL, within the normal range of 0.0–10.0 ng/mL) and radiological complete response (no evidence of disease on CT scan) achieved after the second cycle; the full 4 cycles of adjuvant BEP were completed as scheduled to address the patient’s high-risk profile. During the BEP chemotherapy, she was found to have severe anemia and received four whole blood transfusions during adjuvant treatment.

Two months after finalization of therapy, the patient complained of vaginal bleeding again and AFP was increased to 50.52 ng/ml. Pelvic MRI scan showed a 2.9 x 3.0 x 3.6 cm solid mass near the vaginal cuff which had an indistinct boundary with the posterior wall of the bladder and the anterior wall of the rectum. She was diagnosed with relapse and treated again with BEP regimen. However, after BEP chemotherapy her vaginal bleeding continued and AFP continued to increase (180.70 ng/ml). The chemotherapy regimen was then changed to albumin-bound paclitaxel + carboplatin + bevacizumab. At her last follow-up visit, 17 months after diagnosis, the patient was still without evidence of disease by imaging and tumor markers (AFP = 3.01 ng/ml) (**Figure 1D**).

## Discussion

In this case, preoperative imaging reports showed no obvious lesions in the bilateral adnexal area and pelvic cavity. The postoperative pathology was consistent with a primary cervical tumor with a visible tumor embolus of the lymphovascular space in the cervix, metastasis to a single left external iliac lymph node, and free tumor cells of the lymphatic vascular space in the left ovary. The pathology was consistent with an ovarian metastasis from a primary cervical YST along the lymphatic vessels. We searched for this type of cervical tumor in the SEER database from 2010 to 2019 and found only one other case, noting to have an incidence of 1/56,282.

Similar to ovarian epithelial cancer, the standard treatment for ovarian YST is surgery combined with chemotherapy (15). As ovarian YST is sensitive to BEP chemotherapy, patients with YST have a favorable prognosis, and younger patients could even preserve their fertility at any stage (3, 4). Also, due to its specific biologic behaviors, the treatment strategy of extragonadal YST has changed markedly from radical surgical treatment to conservative surgery combined with chemotherapy, and then to combined chemotherapy alone in special anatomic sites such as the vagina (4). NCCN guidelines recommend radical hysterectomy and concurrent chemoradiation as the standard treatment for cervical cancer. As the rarity of YST in cervical cancer, no similar report or study on the radiosensitivity of HPV negative histology subtypes or less common tumors such as YST have been reported. The patient of our report received neoadjuvant chemotherapy combined with a radical hysterectomy and adjuvant chemotherapy. A recent study investigated the survival outcomes between neoadjuvant chemotherapy and primary debulking surgery in patients with stage III to IV ovarian YSTs or mixed germ cell tumors containing YST elements (16). No statistical difference was found in 3-year disease-free survival and overall survival between the neoadjuvant chemotherapy group and the primary debulking surgery group (log-rank p=0.4 and 0.94) (16). Then, neoadjuvant chemotherapy combined with interval surgery after may be an alternative treatment for patients with advanced YST, especially those with high tumor burden and low performance status.

NCCN guidelines for ovarian germ cell tumor state that patients should receive 3 to 4 cycles of adjuvant BEP chemotherapy to reduce the risk of recurrence (15)—a recommendation extrapolated to our rare cervical YST patient given the absence of site-specific clinical guidelines for cervical YST and the shared biological behavior, chemosensitivity to BEP, and risk stratification principles between gonadal (ovarian) and extragonadal (cervical) YSTs. This 3–4 cycle adjuvant BEP recommendation is specifically indicated for high-risk germ cell tumor patients, and our patient’s multifactorial patient- and disease-related high-risk profile fully justified the administration of 4 cycles of adjuvant BEP chemotherapy (the upper limit of NCCN recommendations) to maximize recurrence risk reduction. The high-risk features included: (1) FIGO stage IIIC1 disease (the most impactful risk factor), confirmed by left external iliac lymph node metastasis; (2) lymphovascular space invasion (LVSI) in both the cervix and left ovary, a well-recognized predictor of metastatic spread and recurrence in gynecologic malignancies; (3) microscopic residual tumor tissues in the exocervix, right sacral ligament, and left ovary after radical cytoreductive surgery; (4) large baseline tumor burden (8 cm cauliflower-like cervical mass, 5.3×2.7×6.4 cm radiologically confirmed mass, 18 mm short-axis metastatic lymph node); and (5) persistently elevated AFP dynamics during neoadjuvant chemotherapy (baseline 512 ng/mL, only reduced to 213.9 ng/mL after 3 cycles of neoadjuvant chemotherapy [1 TC + 2 BEP], far above the normal range of 0.0–10.0 ng/mL).

Another study constructed a risk stratification system based on tumor stage and the interval between treatment and normalization of AFP (17). Patients with FIGO stage I-II and AFP normalization after ≤2 cycles of chemotherapy manifested the best prognosis in both RFS(Recurrence-free survival) and DSS(Disease-free survival) (P < 0.0001)—a stratification that further contextualizes our patient’s risk profile. While our patient achieved AFP normalization and clinical complete remission after the second cycle of postoperative adjuvant BEP chemotherapy (consistent with the ≤2 cycle threshold), she was stratified into the high-recurrence-risk group due to her advanced FIGO stage IIIC1 disease (vs. low-risk stage I-II in the study (17))—a critical stage-related distinction that explains the early recurrence despite guideline-adherent adjuvant BEP chemotherapy and timely AFP normalization.

We performed whole-exome DNA sequencing on the patient’s primary tumor and detected a pathogenic ARID1A nonsense mutation, as well as KRAS and POLE missense mutations (**Table 2**). These genetic alterations are potentially linked to the patient’s early tumor recurrence and BEP chemoresistance: ARID1A loss-of-function and KRAS activating mutations are key drivers of YST progression and platinum resistance (18), while POLE mutation aggravates genomic instability to facilitate tumor recurrence. These molecular findings hold important prognostic value, defining a high-risk molecular profile for the patient that correlates with chemoresistance and early recurrence. They also provide a genetic rationale for the selection of non-platinum salvage therapy (TC + bevacizumab) and the formulation of intensive follow-up strategies, which aligns with the somatic driver alterations and cisplatin resistance-related OVOL2 overexpression reported in YST (18). As no clear alterations could lead to targeted treatment, we searched previous reports for YST second-line treatments. Guidelines recommend TIP (Paclitaxel, ifosfamide, and cisplatin) as the preferred regimen for recurrent germ cell tumors of the ovary, which can reach a complete response rate of 50% (19, 20). This regimen is also effective in patients with poor response to initial chemotherapy or cisplatin-refractory diseases (20). Patients with recurrent metastatic germ cell tumors also may have a good response to high-dose chemotherapy (HDCT) and hematopoietic stem cell transplantation (HSCT) (21, 22). However, our patient refused the above salvage regimen because of a known high incidence of grade 4 myelosuppression reported in TIP regimens and treatment-related death in HDCT. Instead, she chose to receive TC combined with VEGF inhibitor as the salvage regimen.

**Table 2:** Patient’s gene alteration profile.

Gene	Variant types	Exon of variants	cDNA sites	Amino acids variants	Abundance
*ARID1A*	nonsense mutation	20	c.5803G>T	p.Glu1935*	34.06%
*BRD4*	missense mutation	19	c.3869G>A	p.Arg1290His	32.41%
*CTNNB1*	missense mutation	3	c.100_102delinsTGG	p.Gly34Trp	19.31%
*GRM3*	missense mutation	3	c.913G>A	p.Ala305Thr	22.07%
*KMT2D*	missense mutation	38	c.10585G>C	p.Glu3529Gln	10.46%
*KRAS*	missense mutation	5	c.532C>T	p.Pro178Ser	15.71%
*PIK3R1*	in-frame deletion mutation	11	c.1312_1344del	p.Lys438_Lys448del	25.05%
*POLE*	missense mutation	33	c.4276G>A	p.Val1426Ile	45.02%
*RECQL4*	missense mutation	5	c.1051G>A	p.Gly351Ser	38.19%
*SOX9*	frameshift mutation	3	c.710dup	p.Pro238Thrfs*14	25.49%

## Conclusion

YST of the cervix is extremely rare, and the complete response achieved in this patient suggests that TC regimen combined with VEGF inhibitors may be a new option to treat patients with recurrent YST of the cervix.

### Strengths and limitations

This study reports a rare adult case of cervical YST, enriches relevant clinical data, and provides a valuable novel salvage regimen for recurrent/BEP-resistant cases via a complete treatment course. It also conducts systematic literature comparison and adopts a comprehensive clinical evaluation system to ensure data accuracy.

This single-case study has non-generalizable conclusions lacking multi-center verification; homogeneous comparative cases are unavailable and tumor mechanism exploration lacks experimental validation. Follow-up is short, and cervical YST radiosensitivity and the role of radiotherapy remain uninvestigated.

## Data Availability

The raw data supporting the conclusions of this article will be made available by the authors, without undue reservation.
